# Synergistic effects of 6-shogaol and hyperthermia on ACHN renal cancer cells: modulation of ROS and heat shock pro-teins in cancer therapy

**DOI:** 10.3389/fphar.2025.1522285

**Published:** 2025-02-20

**Authors:** Chae Ryeong Ahn, Seung Ho Baek

**Affiliations:** College of Korean Medicine, Dongguk University, Goyang-si, Gyeonggi-do, Republic of Korea

**Keywords:** 6-shogaol, hyperthermia, renal cancer, apoptosis, cell cycle arrest, reactive oxygen species (ROS), heat shock proteins (HSPs), combination treatment

## Abstract

**Introduction:**

Renal cancer is known for its aggressive progression and resistance to standard treatments, underscoring the need for novel therapeutic strategies. This study explores the potential of combining 6-shogaol (6-SHO), a bioactive compound derived from ginger (Zingiber officinale), with hyperthermia to enhance anticancer efficacy in ACHN renal cancer cells.

**Methods:**

ACHN cells were treated with 6-SHO and exposed to hyperthermic conditions. We evaluated the combined effects on apoptosis, cell cycle arrest, and cell proliferation, as well as the role of reactive oxygen species (ROS) and heat shock proteins (HSPs) in mediating these responses.

**Results:**

The combination of 6-SHO and hyperthermia significantly increased apoptosis, induced G2/M phase cell cycle arrest, and reduced cell proliferation more effectively than either treatment alone. ROS played a critical role in these effects, with modulation of HSPs and heat shock factor 1 (HSF1) further disrupting cancer cell survival mechanisms.

**Discussion:**

These findings highlight the synergistic potential of 6-SHO and hyperthermia as a novel therapeutic approach in renal cancer treatment, supporting the need for further research and clinical evaluation.

## 1 Introduction

The incidence of kidney cancer has been steadily increasing worldwide, making it a major health concern in the field of oncology (Cheng et al., 2018; He et al., 2018; Sun et al., 2016; Ahn et al., 2020). Renal cancer, known for its fast progression and considerable resistance to standard therapy, is a significant challenge to both patients and physicians. The search for more effective treatment options has become more pressing, prompting a paradigm shift toward novel approaches that can overcome the limitations of present therapy modalities. This urgency highlights the need for medicines that not only more precisely target cancer, but also address the mechanisms that drive tumor growth, metastasis, and treatment resistance.

Renal cell carcinoma (RCC), the most common type of kidney cancer, is particularly notable for its complex pathophysiology (Allison, 2017; Rini et al., 2019; Soares et al., 2020). The development of RCC is often associated with mutations in the von Hippel-Lindau (VHL) gene, leading to the activation of hypoxia-inducible factors (HIFs) that promote angiogenesis, cell proliferation, and survival (Fenner, 2017; Schmid et al., 2019; Thoma, 2016). Other genetic alterations such as mutations in PBRM1, BAP1, and SETD2 further contribute to the heterogeneity and aggressiveness of the disease. RCC is marked by its ability to invade locally and metastasize early to distant organs, including the lungs, bones, and brain, facilitated by a rich vascular network within the tumor driven by angiogenic factors (Cao et al., 2019; Haibe et al., 2020). Additionally, RCC exhibits resistance to apoptosis and evasion of the immune system, partly through the expression of immune checkpoint proteins such as PD-L1 (Kelsey, 2018).

These pathophysiological aspects complicate the management of renal cancer and highlight the need for novel therapeutic options. Understanding the underlying mechanisms is critical for creating medicines that might better target and manage RCC, perhaps improving patient outcomes.

In recent years, the focus has shifted towards exploring the potential of natural compounds with anticancer properties, among which gingerol, particularly 6-SHO, has garnered significant attention. 6-SHO, a bioactive component found in ginger (Zingiber officinale), has been widely recognized for its broad spectrum of pharmacological effects, including anti-inflammatory, antioxidant, and, most notably, anticancer activities (Hsu et al., 2023; Kim and Lee, 2023; Nina Nina et al., 2024). The anticancer effects of 6-SHO are particularly intriguing due to its ability to induce apoptosis, inhibit cell migration and proliferation, and disrupt the cancer cell cycle across various cancer cell lines (Bawadood et al., 2020; Wozniak et al., 2020; Zhang et al., 2021). These properties make 6-SHO a promising candidate for cancer therapy, including the treatment of renal cancer.

Moreover, the application of hyperthermia as a therapeutic strategy has shown promise in cancer treatment. Hyperthermia involves the controlled application of heat to cancerous tissues and has been found to enhance the efficacy of certain anticancer drugs (Logghe et al., 2024; Qi et al., 2024). The mechanism behind hyperthermia’s effectiveness lies in its ability to induce stress within the tumor environment, leading to the upregulation of heat shock proteins (HSPs) and the activation of various cellular stress responses, which can sensitize cancer cells to chemotherapy and radiation treatment (Yi et al., 2022; Kwon et al., 2023).

Given the potent anticancer properties of 6-SHO and the synergistic potential of combining natural compounds with other therapeutic strategies, such as hyperthermia, there is a compelling case for exploring this combination as a novel approach to renal cancer treatment. This research aims to investigate the synergistic effects of 6-SHO and hyperthermia on renal cancer cells, specifically focusing on their ability to induce apoptosis, inhibit cell proliferation, and affect the cellular stress response mechanisms. By elucidating the molecular and cellular mechanisms underlying these effects, this study seeks to contribute to the development of more effective and targeted therapeutic strategies for renal cancer, addressing the critical need for innovation in cancer treatment.

## 2 Materials and methods

### 2.1 Drugs

6-shogaol (6-SHO) (BP0095, Biopurify, Chengdu, Sichuan, China) is a compound extracted from ginger (Zingiber officinale). Solutions of 6-SHO were prepared at concentrations of 10 and 15 μM using dimethyl sulfoxide (DMSO) acquired from Samchun Chem in Seoul, Korea. These solutions were then stored at a temperature of 4°C until needed.

### 2.2 Cell culture

Regarding the cell culture, the ACHN cell line (Korean Cell Line Bank, Seoul, Korea) was procured. These cells were cultured in DMEM medium, which was enriched with 10% heat-inactivated fetal bovine serum (FBS) and 1% Pen-Strep (10,000 U/mL) (Gibco, Grand Island, NY, United States). The culture conditions were maintained at 37°C in a humidified atmosphere with 5% CO2.

### 2.3 Hyperthermia treatment

For the hyperthermia experiments, ACHN (0.3 × 10^6^ cells) were seeded in 6-well plates, each containing 3 mL of the growth medium. These were then placed in a water bath set to maintain a temperature of 37 or 42°C for a duration of 30 min, unless specified otherwise. The 6-SHO was introduced to the culture at predetermined concentrations 1 hour before the treatment.

### 2.4 MTT assay

The MTT assay was employed to gauge cell proliferation post 6-SHO and hyperthermia exposure. ACHN cells (0.3 × 10^6^ cells) were plated in 96-well plates at a defined concentration and incubated with varying doses of 6-SHO (0, 10 and 15 μM), followed by water bath treatment at either 37 or 42°C in a CO2-enriched atmosphere.

### 2.5 Trypan blue assay

Following Trypan blue (Sigma-Aldrich, St. Louis, MO, United States) staining (0.4%, 1:1 dilution in the cell-containing PBS), the cells’ vitality was measured using a hemocytometer. ACHN cells (0.3 × 10^6^ cells) were planted in 6-well plates, followed by 1 h of 6-SHO treatment and hyperthermia (30 min). After 24 h of post-treatment incubation, cells were collected, diluted 1:4 with PBS, stained, and counted. The cell survival rate was calculated as follows: Cell survival rate is calculated as the ratio of viable cells to total cells multiplied by 100%.

### 2.6 Morphology assay

The morphology assay was used to detect cell proliferation. ACHN cells were planted in a 6-well plate at a density of 0.3 × 10^6^ per well. After adhering to the plates, the cells were treated to 10 and 15 μM 6-SHO for 1 h, then incubated for 30 min at 37 or 42°C. Cells were examined under a microscope and photographed after 24 h (CX-40, Olympus, Tokyo, Japan).

### 2.7 Wound healing assay

ACHN cells were plated in a 6-well plate with a density of 1 × 10^6^ cells per well and stored at 37°C. Once the cells had reached confluence, a thin scratch was made in each well with a yellow pipette tip. Images were taken 0 h (CX-40, Olympus, Tokyo, Japan). After 24 h, the culture material disappeared, the cells were washed with PBS, and additional images were obtained.

### 2.8 Colony formation assay

In a 6-well plate, 400 cells were seeded per well, and the plates were incubated for a full night. The cells were treated with 15 μM 6-SHO for 1 h, then incubated at 37 or 42°C for 30 min for hyperthermia therapy. Following a 2-week period, the cells were stained for 10 minutes at room temperature using a crystal violet solution (Sigma-Aldrich, St. Louis, MO, United States), and then they were cleaned with PBS. Using a standard light microscope (CX-40, Olympus, Tokyo, Japan), images of colonies were captured.

### 2.9 Western blot analysis

Following the extraction from ACHN cells, protein levels were determined. The lysates, post SDS-PAGE separation, were uniformly transferred onto a polyvinylidene difluoride (PVDF) membrane. This membrane was then blocked using TBS that included 0.1% Tween 20% and 5% non-fat milk at ambient temperature. After the blocking phase, the membrane was incubated with various primary antibodies, including anti-caspase-3, anti-caspase-8, anti-caspase-9, anti-survivin, anti-HSP27, anti-HSP70, anti-HSP90, anti-p-ERK (Thr202/Tyr204), anti-ERK, anti-p-p38 (Thr180/Tyr182), anti-p38, anti-p-JNK (Thr183/Tyr185), anti-JNK, anti-p-AKT (Ser473), anti-AKT (Cell Signaling Technology, Danvers, Massachusetts, United States), anti-β-actin, anti-Bcl-2, anti- Bcl-xL, anti-Cyclin B1, anti-Cyclin D1, anti-VEGF, anti-MMP9, anti-MMP2 (Santa Cruz Biotechnology, Inc., Dallas, Texas, United States), as well as anti-HSF1 and anti-pHSF1 from Abcam, including anti-cleaved caspase3 (Genetex, Irvine, California, United States). This incubation occurred overnight at 4°C. Post-incubation, the membranes underwent triple washes with 1x TBS-T and were then exposed to appropriate diluted secondary antibodies (Santa Cruz Biotechnology, Inc., Dallas, Texas, United States) for an hour at room temperature. Following three additional 10-minute washes in TBS-T, the detection was performed using an enhanced chemiluminescence (ECL) technique courtesy of a kit (EMD Merck Millipore, Billerica, MA, United States).

### 2.10 Annexin V assay

The apoptosis ratio was calculated using a Muse^®^ Annexin V and Dead cell kit (Part Number: MCH100105) (EMD Merck Millipore in Billerica, MA, United States). ACHN cells were planted and allowed to adhere overnight (0.3 × 10^6^ cells). ACHN cells were subjected to 6-SHO (1 h) and heat treatment (30 min) and then reacted in a 37° incubator for 24 h. After collecting the cells, 100 μL of AnnexinV and Dead cell reagent was added to each tube according to the instructions provided by the manufacturer, reacted at room temperature for 20 min, and the cells were analyzed using a Muse^®^ Cell Analyzer (EMD Merck Millipore, Billerica, MA, United States).

### 2.11 Mitochondrial membrane potential

To assess the mitochondrial membrane potential, ACHN cells (0.3 × 10^6^ cells), post-treatment, were stained according to guidelines provided by the MitoPotential assay kit (Part Number: MCH100110) (EMD Merck Millipore, Billerica, MA, United States) using prescribed concentrations of MitoPotential working solution and 7-AAD. Analysis was conducted using the Muse^®^ Cell Analyzer.

### 2.12 Cell cycle analysis

The cell cycle phase of ACHN cells (0.3 × 10^6^ cells) in 6-well plates was evaluated after 24 h of co-treatment. After collecting the cells, they were fixed in 70% ice-cold EtOH for an overnight period, washed in PBS, and resuspended in PBS containing 1 mg/mL PI and 10 mg/mL RNase A in a dark room for 10 min. The cell cycle was determined using the Muse^®^ Cell Analyzer (EMD Merck Millipore in Billerica, Massachusetts, United States).

### 2.13 Analysis of reactive oxygen species (ROS)

The production of ROS was measured using a ROS assay kit (Part Number: MCH100111) (EMD Merck Millipore in Billerica, Massachusetts, United States). ACHN cells were treated with an oxidative stress working solution and incubated for 30 min at 37 °C 4 hours after the last treatment. The Muse^®^ Cell Analyzer (EMD Merck Millipore, Billerica, MA, United States) was used to measure ROS levels. Before receiving a 6-SHO and hyperthermia combination treatment, N-acetylcysteine (NAC) was treated for 1.5 h.

### 2.14 Statistical analysis

All numerical values are shown as the mean ± SD. The t-test was used to determine whether the data were statistically significant when compared to the untreated control. *p < 0.05, **p < 0.01; ***p < 0.001.

## 3 Results

### 3.1 Enhanced reduction of ACHN cell growth by combined 6-SHO and 42°C hyperthermia treatment

**FIGURE 1:**
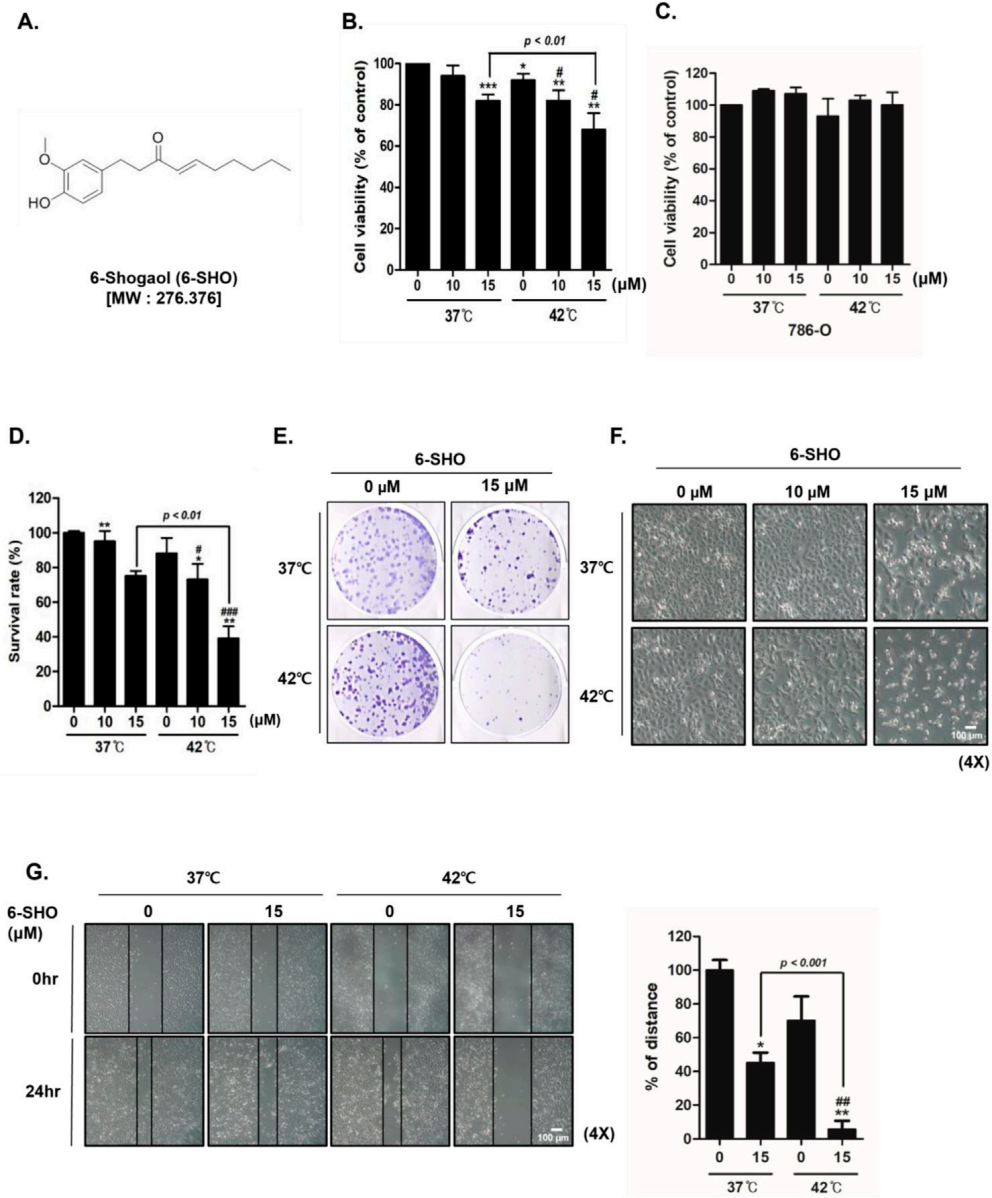
The effect of 6-SHO and hyperthermia co-treatment on ACHN cell viability. ACHN cells were treated with varied 6-SHO concentrations (0, 10, and 15 μM) for a day at 42°C, with or without hyperthermia **(A)** The chemical structure of 6-SHO **(B, C)** MTT was used to determine the percentage of cell viability **(D)** The trypan blue test was administered **(E)** The clonogenic experiment utilized crystal violet staining **(F)** Morphological alterations suggestive of apoptosis were observed under a microscope **(G)** A wound healing test was performed. *p < 0.05, **p < 0.01, ***p < 0.001 vs. control group; #p < 0.05, ##p < 0.01, ###p < 0.001°C vs. 42°C + 0 μM group.

In our study, the dual application of 6-SHO (Figure 1A) with hyperthermia at temperatures of 37°C and 42°C was examined for its effect on ACHN cell proliferation, utilizing MTT assays. Our research showed that combining 6-SHO with a 37°C temperature environment had low effect, while providing 6-SHO at 42°C significantly reduced cell viability at the same quantity (15 µM) (Figure 1B). To assess the specificity of this effect, we performed the same MTT assay on 786-O cells, a normal renal cell line, under identical conditions. The results demonstrated no significant reduction in cell viability in 786-O cells with the combined treatment of 6-SHO and hyperthermia at either 37°C or 42°C, indicating that the observed inhibitory effect is specific to ACHN cancer cells (Figure 1C). The dual treatment markedly increased the proportion of dead cells, as verified by trypan blue assay results (Figure 1D). Moreover, when compared to the dual application of 6-SHO with 37°C, the treatment involving 6-SHO and 42°C temperature substantially reduced colony formation, as illustrated by crystal violet staining in ACHN cells (Figure 1E). Morphological examination further confirmed that the combination of 6-SHO at the tested concentration and elevated temperature effectively hindered the proliferation of cells (Figure 1F). Additionally, a decrease in cell migration was observed in the assay following the co-treatment, indicating a synergistic effect in limiting the proliferative and migratory capabilities of ACHN cells under these conditions (Figure 1G). These results collectively underscore the enhanced effectiveness of combining 6-SHO with elevated temperatures in inhibiting ACHN cell proliferation.

### 3.2 Apoptosis induction in ACHN cells by combined 6-SHO and 42°C hyperthermia treatment

**FIGURE 2:**
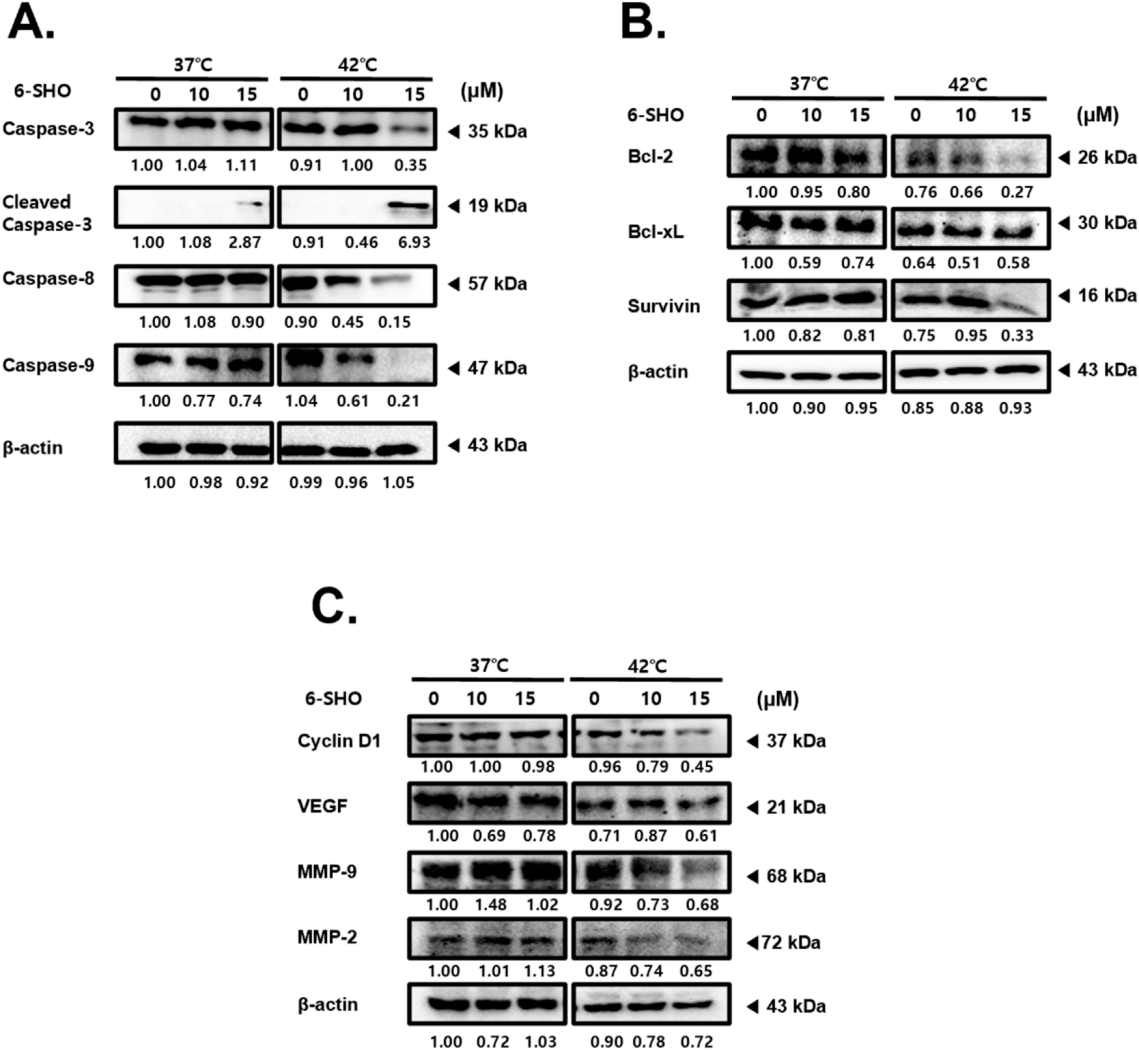
Effect of combined 6-SHO and hyperthermia treatment on apoptosis, cell survival, proliferation markers, and angiogenesis in experimental and control groups. After administering 6-SHO, either with or without hyperthermia, ACHN cells (0.3 × 10^6^) were cultured for a day. Western blot analysis was then done on equal amounts of lysates from whole-cell extracts. Western blots were used to examine the protein expression of **(A)** caspase-3, caspase-8, and caspase-9 **(B)** Bcl-2, Bcl-xL, and survivin, and **(C)** Cyclin D1, VEGF, MMP-9, and MMP-2. β-actin was used as the loading control.

To uncover the mechanisms behind the synergistic effects of 6-SHO and hyperthermia on ACHN cells, we examined the expression levels of various molecules involved in apoptosis, cell growth, metastasis, and angiogenesis. Our results revealed that treating cells with 6-SHO at 42°C notably increases the levels of active caspase-3, a key marker of apoptosis, in a dose-dependent manner, significantly more so than at the standard body temperature of 37°C (Porter and Janicke, 1999; Srivastavaa and Saxena, 2023). The concurrent administration of 6-SHO and 42°C also led to a reduction in caspase-8 and caspase-9 expressions (Figure 2A). Furthermore, this combined treatment significantly decreased the levels of anti-apoptotic proteins within the B-cell lymphoma (Bcl)-2 family, specifically Bcl-2, Bcl-xL, and survivin, also in a dose-responsive manner (Figure 2B) (Kale et al., 2018). Additionally, we explored the expression of Cyclin D1, associated with cell adhesion and migration, along with VEGF, crucial for angiogenesis and cellular movement, and the roles of MMP-9 and MMP-2 in metastasis (Ferrara, 2005; Hosseini et al., 2019; Choi et al., 2023; Reddy et al., 2023). The cotreatment effectively curtailed the metastatic potential and proliferation of ACHN cells by diminishing the levels of Cyclin D1, VEGF, MMP-2, and MMP-9 (Figure 2C). These outcomes suggest that the combination of 6-SHO with hyperthermia impacts multiple cellular pathways, yielding a significant anti-tumor effect.

### 3.3 Enhanced apoptosis and cell cycle arrest in ACHN cells due to 6-SHO and 42°C hyperthermia cotreatment

**FIGURE 3:**
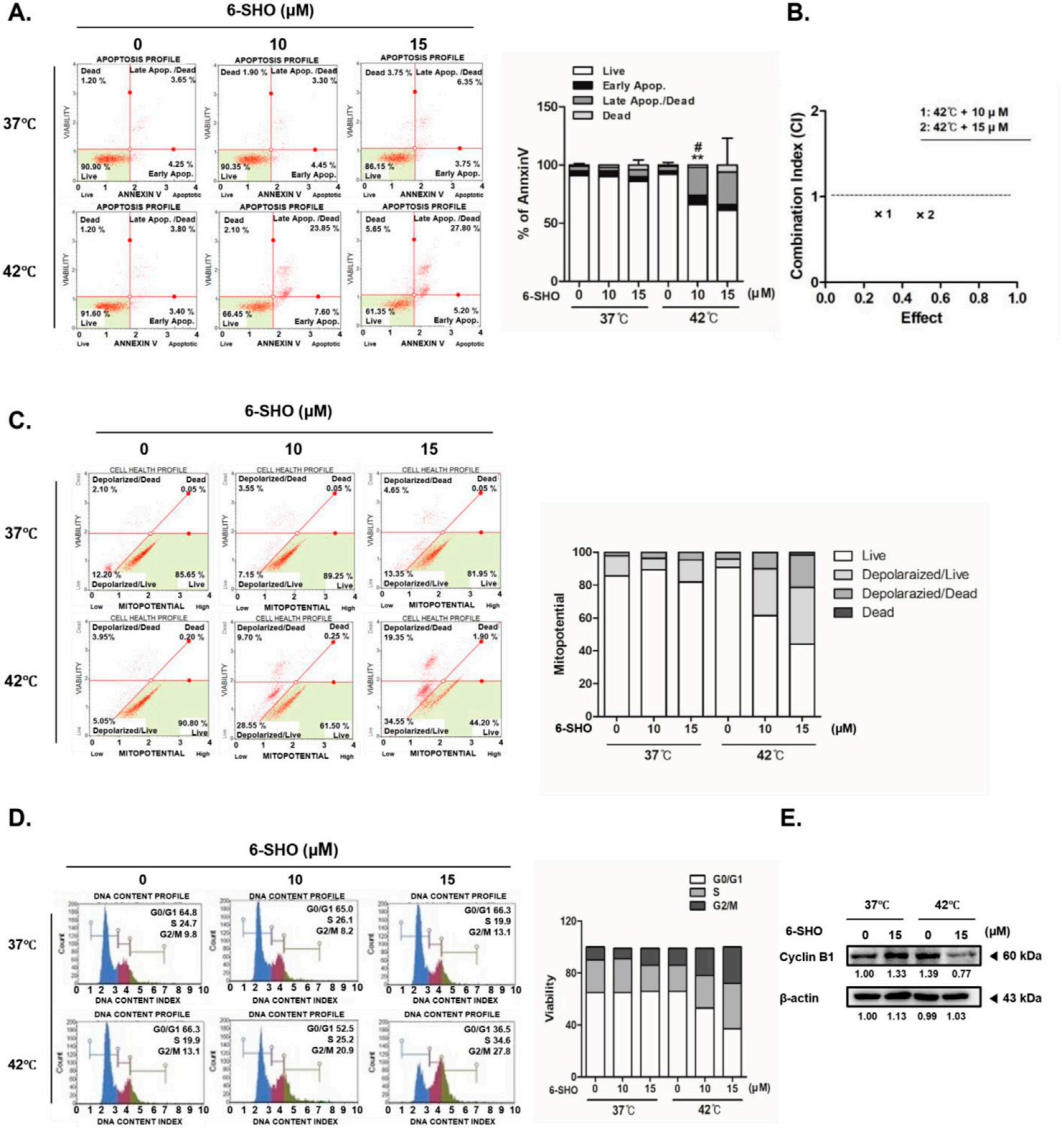
Hyperthermia and 6-SHO modulate the cell cycle and apoptotic processes in ACHN cells. 6-SHO (0 or 15 μM) was tested on ACHN cells (0.3 × 10^6^ cells) with or without hyperthermia **(A)** Apoptosis was recognized using PI staining and Annexin V, and flow cytometry **(B)** Combination index (CI) analysis using the compusyn program **(C)** Mitopotential was measured using a flow cytomet **(D)** The cell cycle and apoptosis profiles were analyzed using flow cytometry **(E)** The expression of cyclin B1 was quantified using a Western blot. β-actin was employed as the loading control.

When we exposed ACHN cells to a combination of 6-SHO and 42°C hyperthermia, there was a more pronounced initiation of apoptosis via annexin V binding than when the cells were treated with hyperthermia alone or with 6-SHO at normal body temperatures (Figure 3A). Significantly, the individual treatments of 6-SHO or hyperthermia did not notably increase the necrotic cell populations (upper left quadrant of the figure). In contrast, the combined treatment significantly raised the apoptotic cell percentage, surging from 10.1% to 33%. To calculate the synergy effect of concurrent treatment, the combination index (CI) was calculated using the compusyn program, and the combination produced a CI value of less than 1, proving that there was a synergistic effect (Figure 3B). Given that the depolarization of the inner mitochondrial membrane potential is a hallmark of apoptosis and cellular distress, we assessed mitochondrial membrane potential changes in ACHN cells (Figure 3C) (Ly et al., 2003; Gottlieb et al., 2003). The results indicated that cotreatment with 6-SHO and 42°C hyperthermia led to a greater increase in cell death compared to the cotreatment at 37°C (21.25% vs. 4.7%), aligning with the annexin V staining outcomes. Furthermore, the cotreatment induced a notable arrest of the cell cycle at the G2/M checkpoint, as shown by flow cytometry analysis (Figure 3D). This effect was corroborated by the observed decrease in cyclin B1 levels in cells treated with 6-SHO under hyperthermic conditions (Figure 3E), suggesting that the strategy of inducing both apoptosis and cell cycle arrest through the cotreatment could play a pivotal role in the anticancer capabilities of this approach.

### 3.4 Enhanced reactive oxygen species generation and MAPK activation by 6-SHO and hyperthermia Co-treatment

**FIGURE 4:**
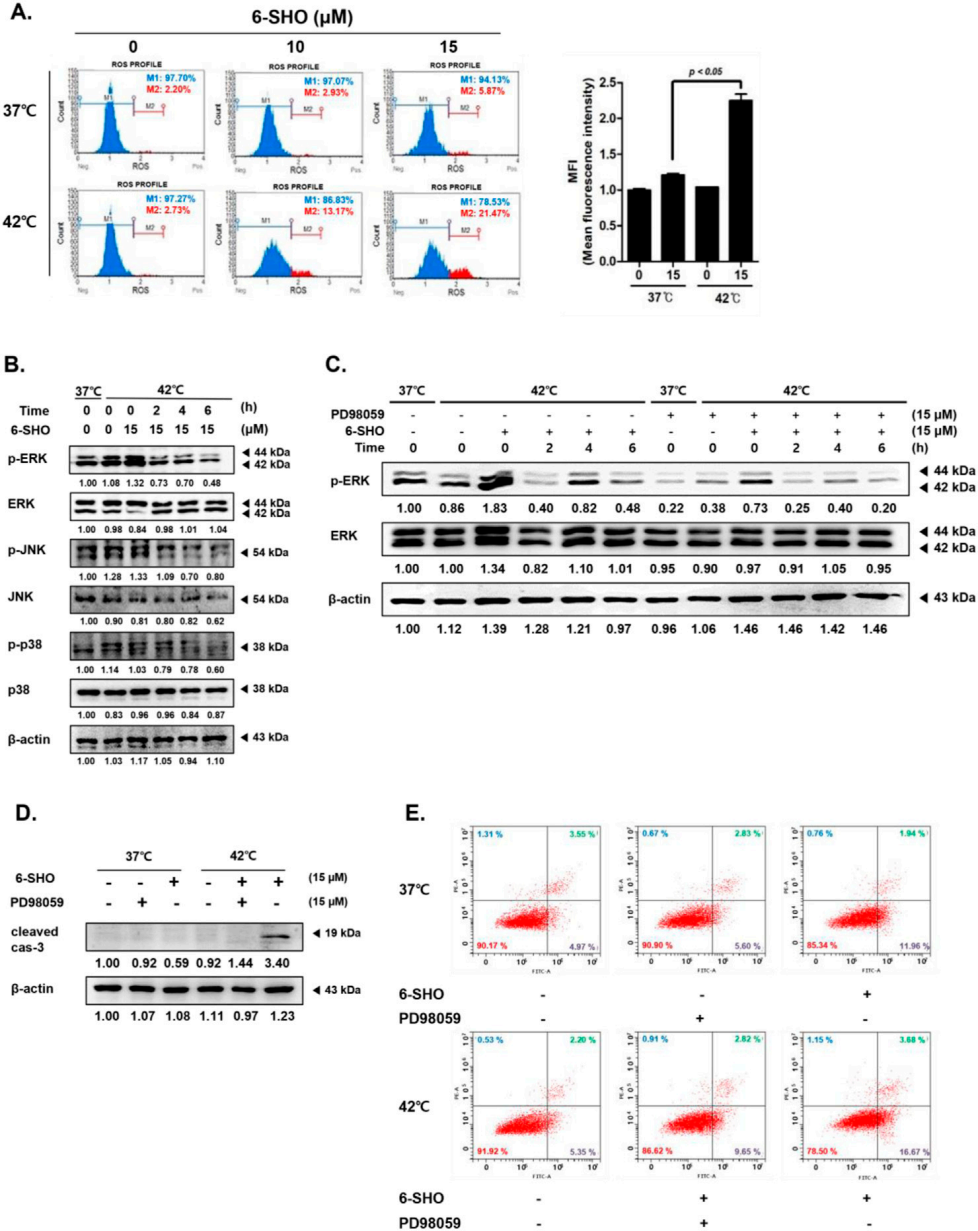
The effects of 6-SHO and hyperthermia on the MAPK signaling pathway and ROS production in ACHN cells. Before undergoing 6-SHO (0 or 15 μM) treatment, either with or without hyperthermia at 42°C **(A)** Flow cytometry was used to evaluate ROS production. **(B)** The levels of p-ERK, ERK, p-JNK, JNK, p-p38, and p38 were determined using Western blot assays. **(C)** The levels of p-ERK and ERK were determined using Western blot assays. **(D)** The expression of cleaved caspase-3 was confirmed by Western blotting with PD98059. The symbols (−) represent the lack of PD98059 or 6-SHO, whereas (+) indicates their presence. **(E)** The analysis employed apoptosis profiling. β-actin was used as a loading control.

Our investigation into the combined effect of 6-SHO and elevated temperature on ACHN cells revealed a significant upsurge in reactive oxygen species (ROS) production, a key factor in apoptosis initiation, when both treatments were applied together (Hua et al., 2016; Trachootham et al., 2009). Through the use of flow cytometry, we established that the synergistic application of 6-SHO with hyperthermia markedly amplified ROS levels beyond what was observed with 6-SHO treatment alone (Figure 4A). Furthermore, we investigated the activation patterns of mitogen-activated protein kinases (MAPKs), including ERK, JNK, and p38, which are involved in apoptosis regulation (Sun et al., 2015; Baek et al., 2016). Elevated ROS levels are known to activate these pathways (Choi et al., 2022). Our results showed that hyperthermia treatment alone activated ERK, and this effect was further enhanced with the addition of 6-SHO, leading to significantly increased ERK phosphorylation (Figure 4B). While JNK and p38 MAPKs also exhibited some degree of activation, their changes were relatively modest compared to the pronounced ERK response. This suggests that the combined treatment predominantly amplifies ERK signaling, which may be key in driving the enhanced apoptotic response observed in NSCLC cells. To investigate the role of ERK in the observed apoptotic effects, we conducted additional experiments using the ERK inhibitor PD98059 (Jeon et al., 2023; Zhao et al., 2024). Western blot analysis demonstrated that PD98059 effectively suppressed the phosphorylation of ERK induced by the dual treatment, confirming its inhibitory effect on ERK activation (Figure 4C). Subsequently, a notable decrease in cleaved caspase-3 levels was observed when the inhibitor was applied, indicating that ERK activation is essential for apoptosis under these conditions (Figure 4D). Furthermore, annexin V assays revealed a significant reduction in apoptosis when PD98059 was used, further supporting the role of ERK in mediating the apoptotic response triggered by the combination treatment (Figure 4E). These findings suggest that enhanced ROS generation and subsequent MAPK activation, particularly through ERK, play a critical role in the apoptosis induced by the combined 6-SHO and hyperthermia treatment.

### 3.5 ROS-dependent apoptotic mechanisms enhanced by 6-SHO and hyperthermia in ACHN cells

**FIGURE 5:**
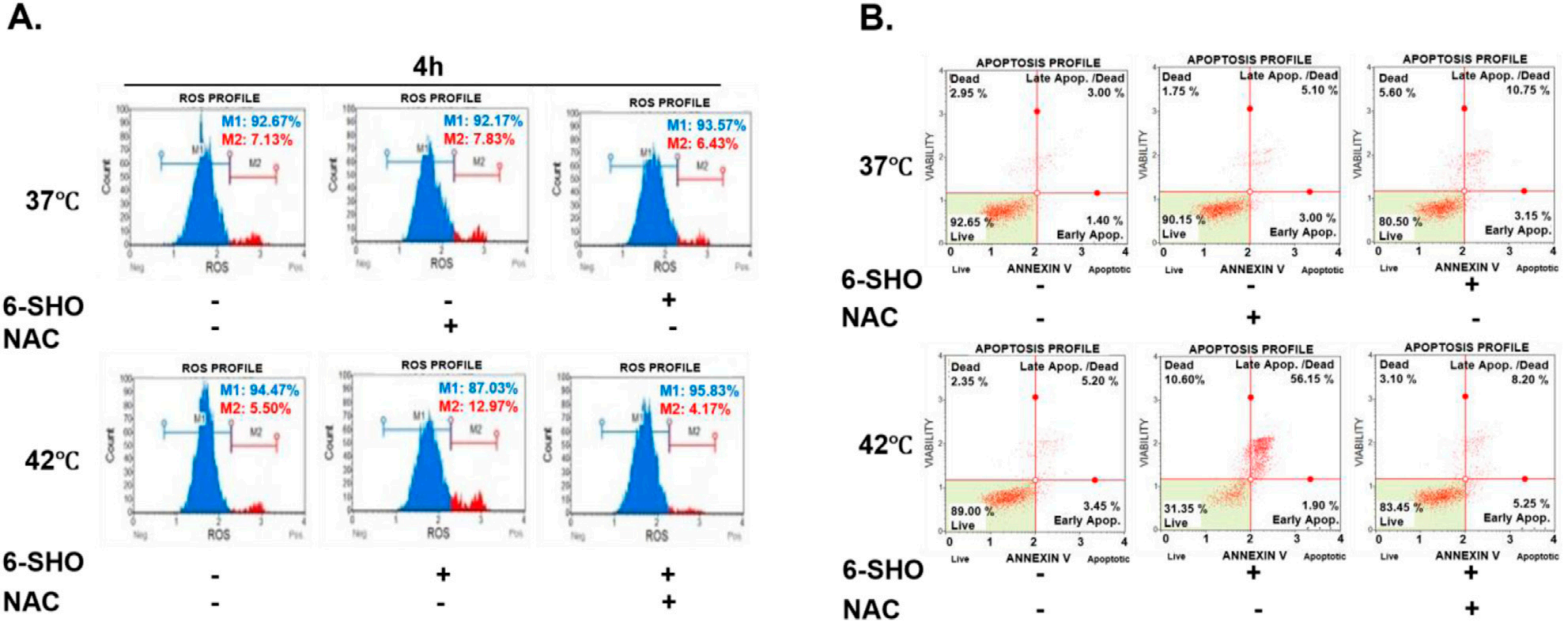
Reduced Apoptosis Induced by 6-SHO and Hyperthermia via ROS Scavenging Mechanisms. ACHN cells were pre-treated with N-acetylcysteine (5 mM) for an hour before being treated with 6-SHO (0 or 15 μM) with or without hyperthermia at 42°C. **(A)** Flow cytometry was used to investigate ROS production. **(B)** The analysis used apoptosis profiling. The symbols (−) indicate the absence of NAC or 6-SHO, whereas (+) shows their presence. β-actin was used as a loading control.

In our analysis of the mechanisms behind the induced apoptosis in ACHN cells treated with 6-SHO and hyperthermia, we first focused on the role of ROS. The increase in ROS production, critical for initiating apoptotic pathways, was significantly attenuated by pretreatment with N-acetylcysteine (NAC), a known ROS scavenger (Zhang et al., 2016; Shimamoto et al., 2011). This led to a marked reduction in ROS levels post co-treatment, as shown in Figure 5A. Subsequent analysis with PI and Annexin V staining revealed a notable decrease in apoptotic cell following NAC pretreatment, thereby emphasizing the pivotal role of ROS in the apoptosis observed with the combined 6-SHO and hyperthermia treatment (Figure 5B).

### 3.6 Inhibition of heat shock response and HSF1 activation by 6-SHO in hyperthermia-stressed ACHN cells

**FIGURE 6:**
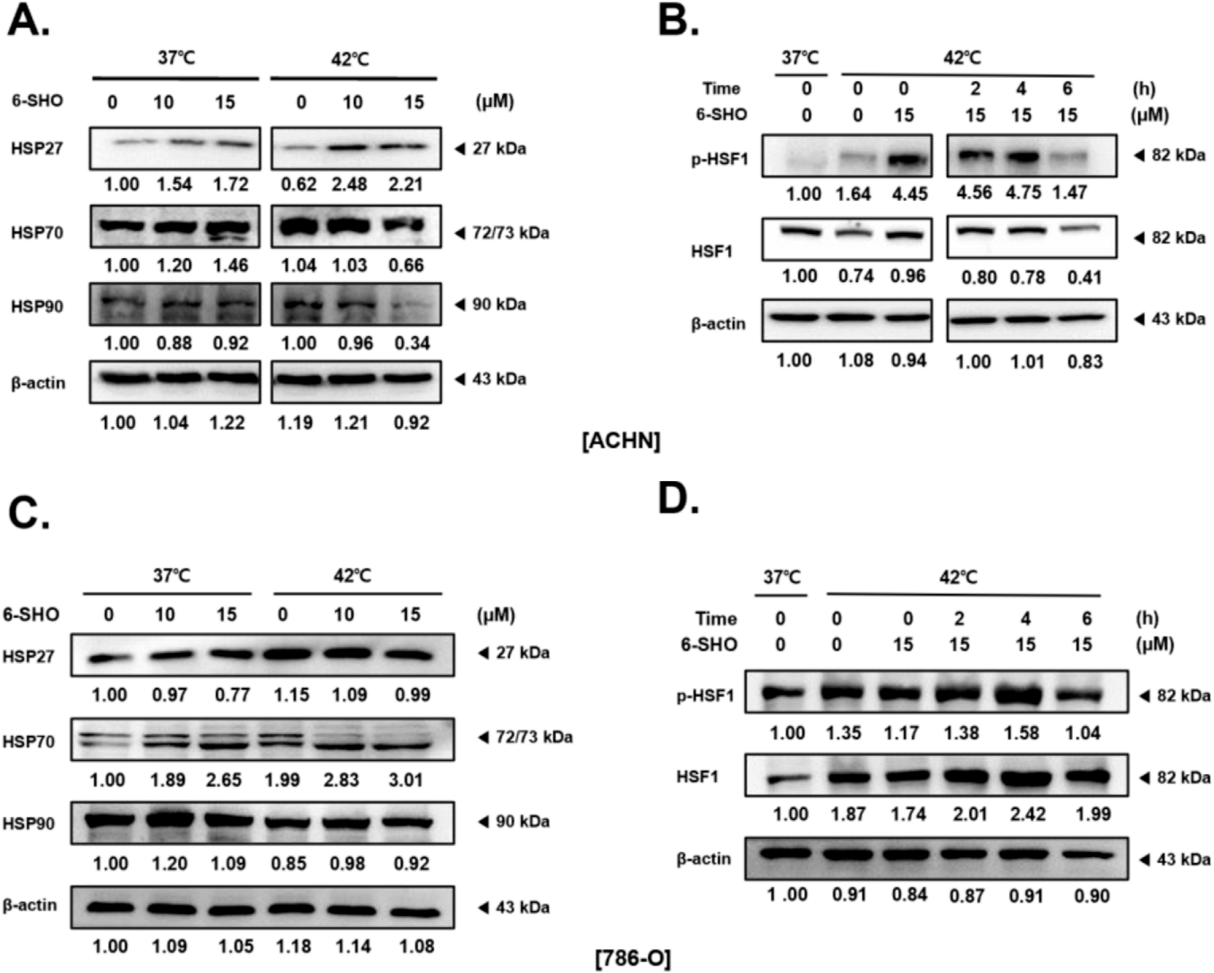
HSF-1 Levels in ACHN and 786-O Cells After Treatment with 6-SHO and Hyperthermia. 6-SHO (0, 10 and 15 μM) was applied to ACHN and 786-O cells (0.3 × 10^6^ cells), with or without hyperthermia. Western blotting was performed to evaluate the protein expression of **(A)** HSP27, HSP70, and HSP90, as well as **(B)** p-HSF1 and HSF, at various times in ACHN cells. **(C, D)** show the results of the same experiments performed in 786-O cells. β-actin was used as a loading control.

The influence of 6-SHO on the expression of heat shock proteins (HSPs) and the activity of heat shock factor 1 (HSF1) in ACHN cells under both normal and heat-stressed conditions was also examined. Heat shock proteins, which serve as molecular chaperones, play vital roles in cell survival, especially under stress (Albakova and Mangasarova, 2021; Aolymat et al., 2023). We observed that hyperthermia at 42°C led to an increase in HSP27, HSP70, and HSP90 levels, whereas 6-SHO treatment noticeably reduced their expression under both normothermic and hyperthermic conditions (Figure 6A). Additionally, while hyperthermia induced the phosphorylation of HSF1, a key regulator of HSP synthesis, this effect was significantly attenuated by the co-treatment with 6-SHO, highlighting its potential to inhibit HSF1 activation and, consequently, HSP synthesis, even after prolonged hyperthermic exposure (Figure 6B). To further assess the specificity of these effects, the same experiments were performed on normal 786-O cells under identical conditions. The results showed that 6-SHO treatment did not significantly affect the expression of HSPs or the activation of HSF1 in 786-O cells under either normothermic or hyperthermic conditions (Figures 6C, D). These findings indicate that the inhibitory effect of 6-SHO on the heat shock response and HSF1 activation is specific to ACHN cancer cells, suggesting a selective mechanism in targeting cancer cells without affecting normal cells.

## 4 Discussion

Renal cancer represents a formidable challenge within the field of oncology, characterized by its aggressive nature and the ability to develop resistance against conventional treatment modalities. The rising global incidence renal cancer underscores an urgent need for the development of innovative therapeutic strategies (Vogelzang and Stadler, 1998; Linehan and Zbar, 2004; Scelo et al., 2016). These strategies must not only effectively target tumor growth and metastasis but also circumvent the prevalent issues of resistance and the limited efficacy that are inherent to current treatment approaches.

6-Shogaol, specifically 6-SHO, a bioactive compound derived from ginger (Zingiber officinale), has attracted significant attention due to its extensive pharmacological effects, which include anti-inflammatory, antioxidant, and, most notably, anticancer activities (Chen et al., 2023; Shahidi et al., 2023). Extensive research has illuminated 6-SHO’s mechanisms of action, revealing its capability to induce apoptosis, inhibit cell migration and proliferation, and disrupt the cancer cell cycle dynamics across a broad spectrum of cancer cell lines (Qi et al., 2015; Wu et al., 2015; Zhang et al., 2022). This has positioned 6-SHO as a particularly intriguing compound for anticancer research, especially in the context of renal cancer therapy.

Hyperthermia, a treatment method that involves heating tumor tissues to 40°C–45°C, has gained prominence as an adjunct therapy in oncology (Crezee et al., 2021; Reinhold and Endrich, 1986). This technique exploits the higher sensitivity of cancer cells to heat compared to normal cells, aiming to selectively damage or kill cancerous tissues while minimizing harm to healthy ones (Issels, 2008; Park and Baek, 2020). The method has shown potential in enhancing the efficacy of traditional treatments like chemotherapy and radiotherapy through mechanisms such as disrupting cancer cell functions and improving drug uptake (Horsman and Overgaard, 2007; Datta et al., 2015; Mortezaee et al., 2021).

Research into hyperthermia has demonstrated its effectiveness in various cancers, suggesting it can lead to better treatment outcomes, especially when used in combination with other therapies (Wust et al., 2002; Ahn et al., 2023). Its role in potentially overcoming drug resistance and reducing treatment-related toxicity further underscores its value as a complementary treatment option in cancer therapy. As ongoing studies continue to explore and optimize hyperthermia’s application, its integration into cancer treatment protocols holds the promise of improving patient outcomes by offering a more targeted and effective approach to cancer care.

Our study further investigates the synergistic effects of 6-SHO in combination with hyperthermia on ACHN cells, which serve as a model for renal carcinoma. This cotreatment approach has demonstrated a marked potency in diminishing cell viability, inhibiting migration, and reducing the capability of the cells to form colonies more effectively than either treatment administered independently. These findings emphasize a multifaceted disruption of cancer cell survival mechanisms, with the enhanced efficacy attributed to the induction of apoptosis and cell cycle arrest at the G2/M phase. Additionally, the treatment significantly increased the percentage of apoptotic cells, as evidenced by PI and Annexin V staining, alongside a notable reduction in cyclin B1 expression, indicating a profound interruption in the cell division processes.

The combined application of 6-SHO and hyperthermia significantly increases ROS production, which plays a crucial role in activating the MAPK pathways. This surge in ROS is essential for the phosphorylation and subsequent activation of the ERK, JNK, and p38 MAPK pathways, leading to a comprehensive attack on the survival mechanisms of cancer cells (Kwak et al., 2023; Wu et al., 2022; Wang et al., 2024). Such an increase in ROS induces a complex cellular response that spans a broader spectrum of apoptotic and survival pathways (Jiang et al., 2024; Navaneethan et al., 2024; Pedrosa et al., 2024; Baek et al., 2015). To further investigate the role of ERK in apoptosis, we employed the ERK inhibitor PD98059 and examined the expression levels of cleaved caspase-3 and annexin V at various time points. The results showed that inhibition of ERK significantly reduced the activation of caspase-3 and the percentage of annexin V-positive cells, confirming the involvement of ERK in apoptosis induction. Additionally, we performed Western blot analysis to monitor ERK activation levels over time. The results demonstrated that the use of the inhibitor significantly suppressed ERK phosphorylation at multiple time points, further supporting its role in mediating the apoptotic response. These findings validate the crucial role of ERK activation in mediating the apoptotic response induced by the combined 6-SHO and hyperthermia treatment. Notably, 6-SHO can act both as an antioxidant and a pro-oxidant depending on its concentration and environmental conditions (Lee et al., 2021; Ribeiro et al., 2018). Under hyperthermic conditions, 6-SHO appears to function primarily as a pro-oxidant, enhancing oxidative stress within the cancer cells (Li et al., 2016). This dual role of 6-SHO contributes to its complex impact on cancer cells, with hyperthermia amplifying its pro-oxidant activity and thereby increasing ROS levels and apoptosis.

To further elucidate the underlying mechanisms of the observed anticancer effects, our research delved into the impact of inhibiting ROS on the efficacy of the treatment regimen. By pre-treating the cells with N-acetylcysteine (NAC), a potent ROS scavenger, we noted a significant diminution in the effectiveness of the 6-SHO and hyperthermia combination in inducing apoptosis (Yedjou and Tchounwou, 2007; Zhitkovich, 2019). This highlights the indispensable role of ROS in mediating the therapeutic effects of the combined treatment, underscoring the importance of oxidative stress in the anticancer activity of 6-SHO and hyperthermia.

Moreover, our study extensively explored the modulation of heat shock proteins (HSPs) and heat shock factor 1 (HSF1) in response to the stress induced by hyperthermia and 6-SHO treatment. HSPs, functioning as molecular chaperones, play a vital role in protecting cells from stress-induced damage, facilitating protein folding, and preventing protein aggregation (Ergul et al., 2020; Zhu and Dai, 2024; Kim et al., 2024). Under the stress conditions induced by hyperthermia, cells typically upregulate the expression of HSPs as a defensive mechanism (Hildebrandt et al., 2002; Singh and Hasday, 2013; Kalamida et al., 2015). Our findings revealed that 6-SHO effectively counteracts this response, leading to a reduction in the expression of HSP27, HSP70, and HSP90, effectively stripping the cancer cells of their protective armor against induced stress. Furthermore, HSF1, the transcription factor that orchestrates the heat shock response by regulating HSP expression, exhibited significantly reduced activation in cells treated with the combination of 6-SHO and hyperthermia, further compromising the cancer cells’ defensive mechanisms against therapeutic stress (Chin et al., 2023; Cyran and Zhitkovich, 2022; Tabuchi and Kondo, 2013).

Interestingly, the specificity of the co-treatment was evidenced by the differential response observed between normal kidney cells (786-O) and renal cancer cells (ACHN). In our experiments, 786-O cells exhibited no significant change in HSP expression following the co-treatment, whereas ACHN cells did. Although hyperthermia caused a transient increase in HSP levels in 786-O cells, no synergistic effect was observed. Cell viability assays (MTT) further confirmed these findings, showing no significant changes in 786-O cells in response to either individual treatments or their combination. Conversely, ACHN cells displayed increased drug toxicity and a marked synergistic effect when subjected to both hyperthermia and the drug. These results strongly suggest that the combined treatment specifically targets ACHN renal cancer cells without adversely affecting normal renal 786-O cells, underscoring its potential for selective anticancer therapy.

By selectively enhancing the anticancer effects in renal cancer cells while sparing normal kidney cells, this co-treatment strategy demonstrates a promising therapeutic approach. This specificity not only maximizes the therapeutic impact on malignant cells but also minimizes potential side effects on normal tissues, addressing one of the critical challenges in cancer therapy.

In addition to the above findings, modulated electro-hyperthermia (mEHT) presents a novel Frontier in the fight against cancer, including renal carcinoma. mEHT employs modulated electromagnetic fields to target and heat cancer cells selectively, offering a more refined approach compared to traditional hyperthermia (Szasz et al., 2019; Viana and Hamar, 2024). This technique not only aims at directly damaging cancer cells but also enhances the immune response against tumors, potentially overcoming the limitations of resistance and side effects associated with conventional therapies (Giunashvili et al., 2024; Minnaar et al., 2020).

The application of mEHT devices in cancer research has demonstrated encouraging outcomes, particularly in enhancing the efficacy of chemotherapy and radiotherapy (Lee et al., 2023). By selectively heating cancer cells, mEHT disrupts their metabolic activities and induces stress responses that make them more vulnerable to therapeutic agents (Minnaar et al., 2022; Petenyi et al., 2021). This integration of mEHT into cancer treatment protocols holds great promise for improving clinical outcomes and patient quality of life.

Moving forward, our research plans to harness the synergistic potential of 6-SHO and mEHT, utilizing our currently available equipment (CPB-2100, dongseo medicare, Inc. Seongnam, Korea), to further elucidate their combined effects on renal cancer cells. The anticipated studies will explore the underlying mechanisms through which this combination enhances cancer cell death, inhibits metastasis, and potentially reduces the adverse effects of current treatment modalities. Given the promising preliminary data, the integration of mEHT into our research represents a significant step towards developing more effective and less toxic therapeutic strategies for renal cancer.

In summary, combining 6-SHO with hyperthermia, and potentially modulated electro-hyperthermia (mEHT), proposes a novel paradigm in renal cancer therapy. This approach exploits cancer cells’ vulnerabilities to oxidative stress and heat shock, enhancing 6-SHO’s anticancer effects and weakening cancer cell defenses. By amplifying 6-SHO’s impact and utilizing targeted heat stress, we pave the way for more effective treatments. Our next steps include conducting animal studies to assess this combination’s efficacy, safety, and side effects, crucial for moving towards human clinical trials. This research is a significant stride towards a new, more effective renal cancer treatment, aiming to improve patient outcomes.

## Data Availability

The original contributions presented in the study are included in the article/Supplementary Material, further inquiries can be directed to the corresponding author.

## Abbreviations

6-SHO, 6-shogaol; DMEM, Dulbecco's Modified Eagle Medium; Bcl, B-cell lymphoma; VEGF, vascular endothelial growth factor; MMP, matrix metallopeptidase; ROS, reactive oxygen species; HSP, heat shock protein; HSF1, heat shock factor 1; MAPK, mitogen-activated protein kinase; ERK, extracellular signal-regulated kinase; JNK, c-Jun NH2-terminal kinase; NAC, N-acetylcysteine; DMSO, dimethyl sulfoxide; MTT, 3-(4,5-dimethylthiazol-2-yl)-2,5- diphenyltetrazolium bromide; FBS, fetal bovine serum; mEHT, modulated electro-hyperthermia.
