# Supplementary material for: Synergistic effects of 6-shogaol and hyperthermia on ACHN renal cancer cells: modulation of ROS and heat shock pro-teins in cancer therapy
Source: Front Pharmacol. 2025 Feb 20;16:1522285. doi: 10.3389/fphar.2025.1522285 (PMC11882530; doi:10.3389/fphar.2025.1522285)
Supplement: Supplementary file 1 [file Presentation1.pptx]

## Slide 1
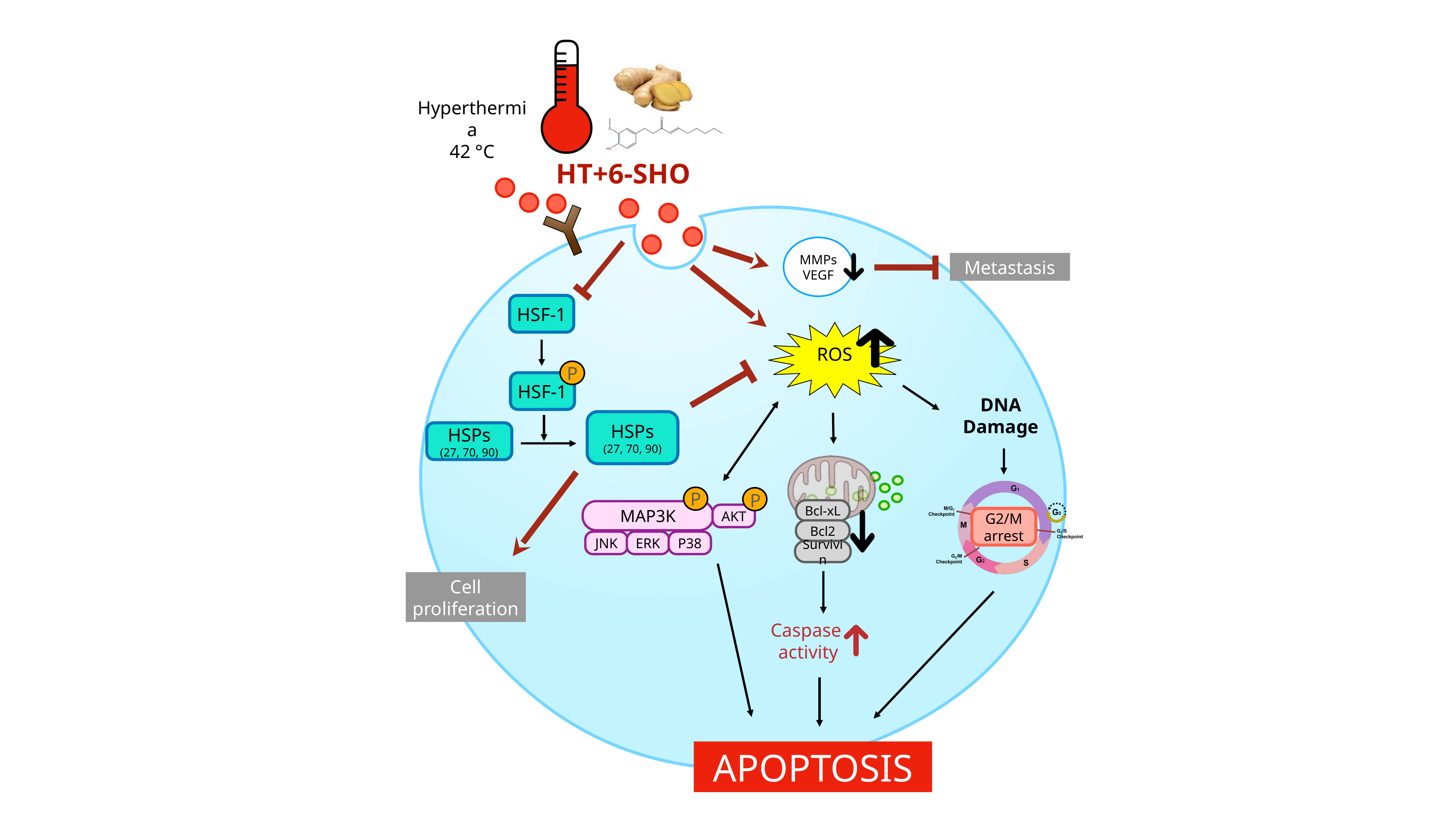

Hyperthermia
42 °C
HT+6-SHO
MMPs
VEGF
Metastasis
HSF-1
ROS
P
HSF-1
DNA
Damage
G2/M
arrest
Bcl-xL
Bcl2
Survivin
Caspase
activity
HSPs
(27, 70, 90)
HSPs
(27, 70, 90)
P
MAP3K
JNK
ERK
P38
P
AKT
Cell proliferation
APOPTOSIS
